# Parent’s experiences of counselling and their need for support following a prenatal diagnosis of congenital heart disease - a qualitative study in a Swedish context

**DOI:** 10.1186/s12884-015-0610-4

**Published:** 2015-08-15

**Authors:** Ewa-Lena Bratt, Stina Järvholm, Britt-Marie Ekman-Joelsson, Lars-Åke Mattson, Mats Mellander

**Affiliations:** Institute of Health and Care Sciences, The Sahlgrenska Academy, University of Gothenburg, Arvid Wallgrens Backe, Box 457, , 405 30 Gothenburg, Sweden; Department of Paediatric Cardiology, The Queen Silvia Childréns hospital, Rondvägen 10, 416 50 Gothenburg, Sweden; Department of Obstetrics and Gynecology, Sahlgrenska University Hospital, Blå Stråket 6, 413 45 Gothenburg, Sweden

## Abstract

**Background:**

Prenatal screening for foetal cardiac abnormalities has been increasingly practiced in Sweden during the last 25 years. A prenatal diagnosis may have medical benefits but may also cause sustained parental psychological distress. The aim of this study was to explore pregnant women’s, and their partner’s, experiences of counselling and need for support during continued pregnancy following a prenatal diagnosis of a cardiac defect. A second aim was to use this information to propose a structured follow-up programme for continued support after the first counselling.

**Method:**

Design: Qualitative study, using interviews performed 5–9 weeks after a prenatal diagnosis of congenital heart disease.

Setting: A tertiary foetal cardiology unit in Sweden

Sample: Six pregnant women and their 6 partners, consecutively recruited after a prenatal diagnosis of an isolated and significant cardiac defect.

Data analysis: Qualitative content analysis.

**Results:**

The analysis resulted in three themes. 1/ Counselling and making a decision - the importance of knowledge and understanding: Short waiting time for specialist evaluation together with clear and straightforward information was essential. Parents called for written information together with a high-quality website with relevant information about congenital heart disease. 2/ Continued support during pregnancy: Continued and easy access to health care professionals, including a paediatric specialist nurse, throughout pregnancy, was important. Contact with couples with similar experiences and social media were also considered valuable sources of support. 3/ Next step – the near future: Practical and economical issues during the postnatal hospital stay and the initial period following the hospital stay were common concerns.

**Conclusions:**

The following aspects should be considered in a structured follow up program during pregnancy after a prenatal diagnosis of CHD; written information, access to a safe web-site with information of high quality in their native language, support from parents with similar experiences and continued contact with a specialist liaison nurse with experience of paediatric cardiology.

## Background

Prenatal screening for foetal cardiac abnormalities, as originally described by Lindsey Allan [1], has been increasingly practiced in Sweden during the last 25 years. The quality of the screening has gradually increased. Today most hospitals use outflow views and the three vessel and tracheal view in addition to the four chamber view of the foetal heart. The purpose of the screening is to optimize perinatal care, especially in duct-dependent lesions, through planning of deliveries to the tertiary centre [2–6]. Prenatal screening also aims to increase pregnant women’s autonomy by informing about the possibility of termination of pregnancy in cases diagnosed before 22 completed weeks.

A prenatal diagnosis of a cardiac defect may cause sustained parental psychological distress, elevated anxiety levels, increased risk for depression and other mental illnesses [7–10]. In addition, maternal stress might affect foetal and child outcomes negatively [11–14]. Thus alterations in foetal growth, neurocognitive development, and cardiovascular health have all been reported to be associated with maternal stress during pregnancy [15–17].

In a recently published scientific statement by the American Heart Association, the importance of counselling and psychosocial support after a prenatal diagnosis of congenital heart disease (CHD) was stressed [18]. Once the foetal diagnosis and its consequences are conveyed, the prenatal health care team should be available to provide emotional support, education and guidance of couples during the time period between the initial prenatal diagnosis and delivery [18]. However, details about the specific content and structure of such a follow-up program from a parental perspective were not highlighted.

The purpose of this study was to (i) explore pregnant women’s and their partner’s experiences of counselling after a prenatal diagnosis of CHD and (ii) to describe their specific needs for support during continued pregnancy.

## Methods

### Setting and participants

Pregnant women and their partners who were referred to our tertiary foetal cardiology centre in western Sweden because of a suspicion of a foetal cardiac anomaly on the screening ultrasound or because of increased risk of foetal cardiac disease, were asked to participate if their foetus was diagnosed with an isolated and significant cardiac defect. A significant defect was defined as one that most likely would require surgical or catheter-based intervention before 1 year of age. An isolated defect was defined as one, which was not associated with extracardiac malformations (normal extracardiac morphology on prenatal ultrasound) or chromosomal aberrations (normal results of amniocentesis). Both the pregnant woman and her partner had to understand and speak the Swedish language. All patients fulfilling these inclusion criteria between July 2013 and March 2014 were consecutively recruited after oral and written information. Information about the study was given during the first visit after the upper gestational age limit for termination of pregnancy, which is 22 + 0 weeks in Sweden. Termination of pregnancy was an exclusion criterion since the purpose of the study was to explore need for support during continued pregnancy. After obtaining informed consent the couple was contacted by phone and asked where and when they preferred to be interviewed. All interviews were performed individually and separate from the partner. The first author (ELB), who was not part of the prenatal health care team, performed all interviews.

The principles of counselling in our unit have been previously described [19]. In short, the parents are counselled by a paediatric cardiologist specialized in foetal cardiology, with full knowledge of the anatomy, physiology, management and current short- and long-term results of postnatal treatment for all types of cardiac defects. The counselling is given in close co-operation with the obstetric team, so that parents are offered information on the exact nature of the problem diagnosed and a full discussion of the available management options at the same time. Later, if appropriate, the counselling may be expanded to include information given by a psychologist, social worker or if needed also a geneticist, neonatologist or paediatric cardiac surgeon. A midwife or nurse is involved from the first counselling visit to the delivery of the baby or termination of the pregnancy to provide continuous support and contact [19].

### Data collection

The interviews, which were guided by open-ended main questions and a set of sub-questions, were audiotaped and transcribed verbatim. During the interview the study participants were invited to talk freely about their experiences and thoughts they had directly after they had received the information about the diagnosis. The median time between the foetal diagnosis and the interview was 7 weeks (range 5–9) and the main questions were; 1/ What were your experiences when given the information about the cardiac diagnosis, its treatment and prognosis? 2/ How did you perceive offered support from the health care professionals? 3/ What kind of support and information did you feel you would need at the time of diagnosis and the following weeks?

### Design and analysis

A qualitative research design was used and the verbatim-transcribed interviews were analysed using qualitative content analysis. This is a method for the systematic analysis of interview texts in various steps [20, 21]. The content analysis used in this study focused on the manifest content, which emphases on what the text says and deals with and describes the visible, obvious components [20, 21]. During the analysis, when identifying and describing the manifest content, efforts were made to stay close to the interview text. First, the interviews were combined into one text and were then read several times to get an initial understanding of the whole. Second, sentences of importance were identified and highlighted (meaning units). Third, the meaning units were condensed into shorter units still preserving the core content. Fourth, the condensed meaning units were coded and labelled on the basis of shared concepts. Fifth, the codes were compared to identify similarities and differences and nine subthemes were developed based on the codes (what the text communicated). Sixth, a comparison and interpretation of the subthemes were undertaken, from which the three themes were developed (Table 2).

The first and second authors (ELB, SJ) continuously discussed the coding of the text, the classification of the sub-themes as well as the creation of the themes.

### Ethical approval

The study was approved by the regional Ethical Review Board in Gothenburg (Study Code 710-12) and performed according to the Helsinki declaration [22]. Written and verbal information about the study was given, stressing that participation was entirely voluntary, and informed consent was obtained. Confidentiality was guaranteed.

## Results

Fourteen prospective parents were invited to participate. Six mothers and six fathers (six couples) accepted the invitation (Table 1). Two individuals declined and their sociodemographic and medical characteristics did not differ from the participating families except that they were the only couple that already had a child with CHD. A cardiac anomaly was suspected during the routine ultrasound screening examination in five of the six foetuses and in the remaining foetus during examination in association with a course in foetal cardiology. The median gestational age at the first specialist foetal echocardiographic examination was 18 weeks (range 17–22). The diagnoses were a/ hypoplastic left heart syndrome in two cases, b/ corrected transposition, c/ tricuspid atresia with interrupted aortic arch, d/ pulmonary atresia with intact ventricular septum and a severe tricuspid regurgitation and e/ pulmonary atresia with a ventricular septal defect and major aortico-pulmonary collaterals in one each of the remaining four cases.

**Table 1:** Sociodemographic data - Key variables of the participants *n* = 12. Numbers, median and (ranges)

Age	33 (24–37)
Females/males	6/6
No previous children	5
Years in present relationship	8.4 (4–15)
Educational level	
- Primary school	0
- Upper secondary school	1
- University	9
- Other	2
Living in a city with	
>200 000 inhabitants	8
50000–200000	2
<50000	0
Rural area	2

In all, 12 interviews were conducted. The interviews were performed individually in the patient’s home (*n* = 4), at her/his workplace (*n* = 2) or at the hospital (*n* = 6) and always by the same person (ELB). The median interview duration was 36 min (range 20–76min).

The analysis resulted in three themes: “Counselling and making a decision - the importance of knowledge and understanding”, “Continued support during pregnancy” and “Next step – the near future” (Table 2). Quotes will be referred to F as for father or to M as for mother and the sequence number of the interview is given.

**Table 2:** Themes and subthemes

1. Counselling and making a decision - the importance of knowledge and understanding	1:1 The need to know
	1:2 The need to make a decision - consideration of termination of pregnancy
	1:3 The need to have more information
2. Continued support during pregnancy	2:1 Support from professionals
	2:2 Support from family and friends
	2:3 Support from those in the same situation
3. Next step – the near future	3:1 The delivery
	3:2 Practical issues
	3:3 The daily life for the child/children

### Counselling and making a decision - the importance of knowledge and understanding

#### The need to know

It was obvious that most prospective parents were expecting a normal result from the routine scan. They expected to leave the routine scan with an ultrasound image of their foetus and a more precise date for delivery. When a suspicion was raised of a cardiac problem a minimal time delay to a specialist examination was considered to be crucial.

*“What made it “bearable”, was partly because we got an appointment with the specialist after such a short time and then it proceeded really fast to the next … till we got to do an amniocentesis . So it was not so long we had to wait…” F 3*

### The need to make a decision – consideration of termination of pregnancy

The prospective parents experienced this period as being very stressful. They wished to evaluate all available information, including the degree of uncertainty of the diagnosis and the results they could expect from postnatal treatment including the likely long-term outcome and quality of life in survivors, before deciding to continue or to terminate the pregnancy. This process was described as one of the most stressful situations experienced in life. The short time available (in some cases just a few days) for the decision contributed to the stress and it was described as very traumatic to have to make a decision about life and death for their foetus. Most parents considered the results of the karyotype to be crucial for their decision.

*“…we felt that we could not do it…// it is nonetheless a life and it is our child…this was not a decision I was supposed to make, as a human…this could not be the intention…that I should have to make such a decision when the pregnancy had continued for so long…” M6*

### The need for more information

Following the foetal cardiologist consultation prospective parents were very active to seek information by themselves on the Internet, sometimes already before they left the hospital. There they found information about the diagnosis, treatment, complications, morbidity, mortality, daily life issues and quality of life. Parents also called for written information in a leaflet and they requested a high quality website with information about the specific CHD which had been diagnosed in their own foetus. Their ambition to increase their knowledge and understanding of CHD did not solely focus on medical information. They also wanted to learn about daily life issues, such as functioning in school, quality of life and practical details that might be of importance for the child’s future life. Meaningful information was also found on the internet in private blogs written by parents of children with CHD and by young people with CHD. Thus, social media played an important role; parents found comfort in reading other personal stories on the internet. These experiences gave them insight into the daily life of people in similar situations with first hand experience. They seemed to be aware of the risk that these stories might sometimes mainly describe negative experiences. However, a majority of the parents were attentive and filtered the information.

*“Well … it would have been nice if when I want to check on the Internet I would know that the information is correct.” M 1*

*“… I took up the cell phone and searched on the Internet directly… “no outflow of the left ventricle and ultrasound” and then got, like, the worst possible scenarios…” M4*

They also emphasized that oral information needed to be repeated several times at different occasions. The information gathered from different sources after the first counselling and the increasing understanding led to new questions but also to a need for confirmation that they got it all right.

*“I always have a very hard time (sigh) thinking of things in processes and so otherwise, but in this I have really been like that and taken it a bit little by little and not that everything has to happen at once.” F6*

### Continued support during pregnancy

#### Support from professionals

The prospective parents expressed a wide-range of support needed in order to be able to handle the situation. These included contact with a social worker and a paediatric cardiology nurse specialist.

*“When we feel we want to discuss anything regarding the cardiac defect we would call the foetal cardiologist or the paediatric cardiac nurse. We would not call the midwife for that.”F9*

Parents also described that certain health care professionals were more important than others during certain periods. Both the foetal cardiologist and the obstetrician were important to them. The paediatric nurse became increasingly important as the pregnancy proceeded. Some parents wanted contact with a social worker from the start whilst others preferred to have this only later in pregnancy. Continuous and easy access, throughout the rest of pregnancy, to health care professionals was considered important.

### Support from family and friends

Support from family and friends were also important. Some parents were more active than others in seeking support. Needs appeared to differ from couple to couple, between partners and from one time-point to another during pregnancy.

*“…we have had … my mom and also my stepfather, or both of our families have been very supportive, but I can feel that especially my mom has probably been very … almost like a psychologist, that is we have talked a lot. She has read much herself, almost more than we have managed to do ourselves, and been very encouraging. She has pointed out that we must accept what we have, and see what we can do about it, that we have to fix this now. We have had very, very good support from both families of course.”*

*“I believe in repetition, and also asking every time…how do you feel now? // Because we take in a lot but much of it disappears …”F8*

### Support from those in the same situation

It was clear that sharing experiences with and getting support from others who had been in a similar situation was crucial. Offering contact with other couples was part of the routine from the start of the study and this was highly appreciated. The prospective parents also felt support and comfort in reading private blogs because it gave them further insight in to what kind of life they could expect.

*“We have met another couple who are in the same situation, and I think it’s great to hear a lot about other people’s stories and experiences surrounding it all. I realize of course that each case is different so I can’t draw any major conclusions but still…” M11*

*“But immediately after // I was very uplifted by these blogs, for there were so many who described that they had a really horrible first time, but that it later became good, actually, and that their children were normal. I was extremely glad to see all these girls and boys who lived normal live…”F2*

### Next step – the future

#### The delivery

Concerns related to the pending delivery and initial stay at the children’s hospital was sometimes a distress. Parents wanted to be assured that the medical information of importance regarding their foetus/child was accessible for the staff in charge during the delivery. This insight reinforced their feeling of security. Detailed information about the expected sequence of events after delivery was appreciated. Such information included, whether parents would be allowed to hold the baby after delivery or if he or she would be taken directly to the intensive care unit, if it would be possible to breastfeed and also more practical questions such as if the baby can wear private clothes, which family members can visit, and whether we as parents can sleep in the same room as our baby?

*“Will breastfeeding be possible? Where will the baby be? Will it be possible for us to be with her? Of course you think about stuff like that.” M4*

### Practical issues

Practical and economical issues during the initial hospital stay and the period immediately after discharge was a concern. Details such as if the parents would be allowed to stay in the hospital with their child, issues addressing parental leave, sick leave and the possible financial burden as a result of their child’s disease were common questions.

*“Or when you will be away from work, how it is solved. So you don’t have to worry about economics. This is actually a trifle in the whole context, // … it’s something that you want to forget and therefore have it resolved as quickly and smoothly as possible.” F9*

Couples who visited the paediatric cardiac ward during pregnancy experienced this as an advantage. The upcoming stay in the paediatric cardiac ward was no longer completely unfamiliar.

*“…A relief to be familiar with the environment… the more you know on beforehand… before our own daughter will be there, the better it is …”F4*

### The daily life for the child/children

Thoughts and worries addressing the future daily life were common. These concerns included, the child’s quality of life and physical capacity. Parents with previous children were worried about how sibling(s) would react and how they could be prepared and how the family situation would be after the baby was born. Most parents appreciated if the social worker had experience from a paediatric setting including knowledge about how it is to live with a child with a chronic condition.

*“So, then I did a Google search… then … we sat there … and we saw that there was one who had been operated upon 19 times during 3 years and one who was running three miles a day. Neither me nor my wife do that so then I thought that maybe it is not so …bad” M4*

*“… will the child be able to do all the normal things, if you say, out and run and bike and stuff like that // … will he be able to do virtually all the normal things more or less.” She might not be a top performing athlete, but it is not the most important thing in the world either.” F8*

## Discussion

This study, similar to previous studies on prenatal screening, showed that parents were unprepared to handle the information that their foetus had a cardiac defect. Since the scan was offered routinely it was viewed as non-threatening [23–25]. After confirmation of the diagnosis couples have to determine, under the pressure of a limited time period, whether the pregnancy should continue or be terminated. Counselling and support during this period requires a high degree of professionalism, especially by those who perform the foetal echocardiographic examination and provide information about the diagnosis [26]. The health care professionals involved must have knowledge about treatment options, be up-dated on the latest results of postnatal treatment in the short and long term, risk of complications, quality of life as well as psychosocial consequences for the child with CHD and for the family [18].

To receive information about CHD in the foetus is extremely stressful for the prospective parents who are expected to understand treatment options and their results as well as many other implications of having a child with CHD. For most parents medical terminology is unknown and may be a challenge [27] even if questions can be asked directly to the foetal cardiologist and the obstetrician during the counselling. Supplementary written information was considered useful by several of the subjects. It enhanced recall and understanding and was helpful when the problem later had to be explained to significant others. This was in line with results reported in previous studies [24, 28]. When parents searched for additional information on the Internet, it was often difficult to find information in Swedish. To find relevant information using various Web search engines was described as a challenge similar to the results of the study by Carlson et al. [29]. Part of this challenge was the foreign language and translation of relevant information would be helpful. Expectant couples can be overwhelmed by all information found on the Internet and sometimes it may be difficult to sort out relevant data. Recommendation from the profession regarding specific, reliable websites may help [27] and is also emphasized in a previous study from Sweden [28]. Except for medical information, the Internet also provides information about daily life from persons with first hand experience. Even though subjective and personal it can be helpful for some individuals to share experiences of other parents in the same situation. Parents also sought direct contact with couples in the same situation. For some couples this was an important way of support and should be considered to be implemented as an option in a structured follow-up program.

A well-functioning collaboration between nurses and physicians in the antenatal care and paediatric cardiology units is desirable to optimize psychosocial support. Early contact with a paediatric cardiac nurse coordinator (liaison nurse) was also appreciated. This should be an experienced nurse who could answer questions with confidence regarding not only the immediate postnatal period but also practical issues about postoperative follow-up and daily life.

In this study parents considered a social worker with experience from the paediatric setting to be the most suitable to give advice about how to handle information to siblings and also about financial issues such as parental leave and sick leave.

### Study limitations

This was a single centre study restricted to patients with Swedish as their native language. The majority of the study population were highly educated, which might have had an impact on the results. The result may therefore not be applicable to other groups of patients. We did not include patients who decided to terminate the pregnancy. Although this may be considered a limitation, the main purpose of the study was to increase our understanding of the needs of prospective parents during continued pregnancy. It might well be that couples deciding to terminate pregnancy experience counselling differently but this should be the subject of a separate study.

## Conclusion

Women and their partners valued a short waiting time from the first suspicion of foetal heart disease to the final diagnosis and counselling. Continued support throughout pregnancy was considered important emphasizing the value of web-based information of high quality, written information, support from parents with similar experiences and continued contact with a specialist liaison nurse. These results should be helpful when designing a structured follow up programme for prospective parents after a prenatal diagnosis of CHD.

## References

[1] Allan LD, Sharland GK, Milburn A, Lockhart SM, Groves AM, Anderson RH, Cook AC, Fagg NL (1994). Prospective diagnosis of 1,006 consecutive cases of congenital heart disease in the fetus. J Am Coll Cardiol.

[2] Morris SA, Ethen MK, Penny DJ, Canfield MA, Minard CG, Fixler DE, Nembhard WN (2014). Prenatal diagnosis, birth location, surgical center, and neonatal mortality in infants with hypoplastic left heart syndrome. Circulation.

[3] Bonnet D, Coltri A, Butera G, Fermont L, Le Bidois J, Kachaner J, Sidi D (1999). Detection of transposition of the great arteries in fetuses reduces neonatal morbidity and mortality. Circulation.

[4] Eapen RS, Rowland DG, Franklin WH (1998). Effect of prenatal diagnosis of critical left heart obstruction on perinatal morbidity and mortality. Am J Perinatol.

[5] Kipps AK, Feuille C, Azakie A, Hoffman JI, Tabbutt S, Brook MM, Moon-Grady AJ (2011). Prenatal diagnosis of hypoplastic left heart syndrome in current era. Am J Cardiol.

[6] Tworetzky W, McElhinney DB, Reddy VM, Brook MM, Hanley FL, Silverman NH (2001). Improved surgical outcome after fetal diagnosis of hypoplastic left heart syndrome. Circulation.

[7] Kaasen A, Helbig A, Malt UF, Naes T, Skari H, Haugen G (2010). Acute maternal social dysfunction, health perception and psychological distress after ultrasonographic detection of a fetal structural anomaly. BJOG.

[8] Leuthner SR, Bolger M, Frommelt M, Nelson R (2003). The impact of abnormal fetal echocardiography on expectant parents’ experience of pregnancy: a pilot study. J Psychosom Obstet Gynaecol.

[9] Skari H, Malt UF, Bjornland K, Egeland T, Haugen G, Skreden M, Dalholt Bjork M, Bjornstad Ostensen A, Emblem R (2006). Prenatal diagnosis of congenital malformations and parental psychological distress--a prospective longitudinal cohort study. Prenat Diagn.

[10] Rosenberg KB, Monk C, Glickstein JS, Levasseur SM, Simpson LL, Kleinman CS, Williams IA (2010). Referral for fetal echocardiography is associated with increased maternal anxiety. J Psychosom Obstet Gynaecol.

[11] Glover V (2014). Maternal depression, anxiety and stress during pregnancy and child outcome; what needs to be done. Best Pract Res Clin Obstet Gynaecol.

[12] Talge NM, Neal C, Glover V, Early Stress TR, Prevention Science Network F, Neonatal Experience on C, Adolescent Mental H (2007). Antenatal maternal stress and long-term effects on child neurodevelopment: how and why?. J Child Psychol Psychiatry.

[13] Brunton PJ (2013). Effects of maternal exposure to social stress during pregnancy: consequences for mother and offspring. Reproduction.

[14] Glover V (2015). Prenatal stress and its effects on the fetus and the child: possible underlying biological mechanisms. Adv Neurobiol.

[15] Mulder EJ, de Medina PG R, Huizink AC, Van den Bergh BR, Buitelaar JK, Visser GH (2002). Prenatal maternal stress: effects on pregnancy and the (unborn) child. Early Hum Dev.

[16] Weinstock M (2005). The potential influence of maternal stress hormones on development and mental health of the offspring. Brain Behav Immun.

[17] Maina G, Saracco P, Giolito MR, Danelon D, Bogetto F, Todros T (2008). Impact of maternal psychological distress on fetal weight, prematurity and intrauterine growth retardation. J Aff Disord.

[18] Donofrio MT, Moon-Grady AJ, Hornberger LK, Copel JA, Sklansky MS, Abuhamad A, Cuneo BF, Huhta JC, Jonas RA, Krishnan A (2014). Diagnosis and treatment of fetal cardiac disease: a scientific statement from the American Heart Association. Circulation.

[19] Mellander M (2005). Perinatal management, counselling and outcome of fetuses with congenital heart disease. Semin Fetal Neonatal Med.

[20] Graneheim UH, Lundman B (2004). Qualitative content analysis in nursing research: concepts, procedures and measures to achieve trustworthiness. Nurse Educ Today.

[21] Krippendorff K (2004). Content analysis : an introduction to its methodology.

[22] World Medical Association. Declaration of Helsinki. Ethical Principles for Medical Research Involving Human Subjects. In.: The World Medical Association; 1964.

[23] Lalor J, Begley CM, Galavan E (2009). Recasting Hope: a process of adaptation following fetal anomaly diagnosis. Soc Sci Med.

[24] Lalor JG, Devane D, Begley CM (2007). Unexpected diagnosis of fetal abnormality: women’s encounters with caregivers. Birth.

[25] Sommerseth E, Sundby J (2010). Women’s experiences when ultrasound examinations give unexpected findings in the second trimester. Women Birth.

[26] Killen SA, Mouledoux JH, Kavanaugh-McHugh A (2014). Pediatric prenatal diagnosis of congenital heart disease. Curr Opin Pediatr.

[27] Hilton-Kamm D, Sklansky M, Chang RK (2014). How Not to Tell Parents About Their Child’s New Diagnosis of Congenital Heart Disease: An Internet Survey of 841 Parents. Pediatr Cardiol.

[28] Carlsson T, Bergman G, Melander Marttala U, Wadensten B, Mattsson E (2015). Information following a Diagnosis of Congenital Heart Defect. Experiences among Parents to Prenatally Diagnosed Children. PLoS One.

[29] Carlsson T, Bergman G, Karlsson AM, Mattsson E (2015). Content and quality of information websites about congenital heart defects following a prenatal diagnosis. Interact J Med Res.

